# Gout/hyperuricemia reduces the risk of Alzheimer's disease: A meta‐analysis based on latest evidence

**DOI:** 10.1002/brb3.3207

**Published:** 2023-09-04

**Authors:** Long Wang, Zheng Tan, Fu‐Yu Wang, Wen‐Pei Wu, Jun‐Cang Wu

**Affiliations:** ^1^ Department of Neurology Hefei Hospital Affiliated to Anhui Medical University (The Second People's Hospital of Hefei) Hefei Anhui China; ^2^ Graduate school The Fifth Clinical Medical College of Anhui Medical University Hefei Anhui China; ^3^ Department of Pharmacy The Second People's Hospital of Hefei Hefei Anhui China

**Keywords:** Alzheimer's disease, association, gout, hyperuricemia, risk

## Abstract

**Objective:**

Previous studies have found the potential role of gout or hyperuricemia in subsequent development of Alzheimer's disease (AD) but reported inconsistent results. We conducted the current meta‐analysis to evaluate whether an association exists between gout/ hyperuricemia and AD.

**Methods:**

We systematically searched PubMed and EMBASE for the published cohort studies that measured the risk of AD in subject with gout/ hyperuricemia up to May 20, 2023. Data extraction was employed by two authors independently. Rev Man 5.3 and Stata 15.0 software were used to calculate the relative ratio (RR) or hazard ratio (HR) for including studies. Subgroup analysis was performed to assess the sources of heterogeneity. A random‐effects model was adopted when heterogeneity was present. The funnel plot, Begg's test, and and Egger's test were used to assess publication bias.

**Results:**

After rigorous screening, seven eligible studies were included in the final analyses. Pooled results indicated that gout or hyperuricemia decreases the risk of AD (RR: 0.69, 95% CI: 0.64∼0.72), with a high heterogeneity of 93%. Subgroup analyses showed that regional distribution was the source of heterogeneity. Egger's and Begg's tests as well as visual inspection of funnel plot suggested no publication bias in the studies.

**Conclusion:**

The findings suggested that gout or hyperuricemia might have a protective effect against AD. This negative correlation should be verified by more cohort studies due to the existence of substantial heterogeneity.

## Graphical abstract

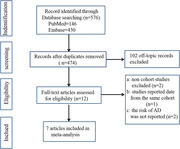

## 1 INTRODUCTION

Dementia is common neurological diseases affecting elderly subjects. Existing epidemiological surveys indicated that approximately 50 million people worldwide were living with dementia, projected to triple in 2050 (Sarkis et al., 2019). Alzheimer's disease (AD) is a complex, irreversible and progressive neurodegenerative disorder of the brain, which is the most frequent form of dementia, a general term for persistent memory decline, learning and other cognitive dysfunction that interfere with daily life (Alzheimer's Disease Facts & Figures, 2021). However, there is no valid treatment for AD (Passeri et al., 2022). Therefore, it is essential to investigate the pathogenesis and potential risk factors of AD to improve the prevention and treatment of its onset and progression.

Gout is a systemic inflammatory disorder characterized by the formation of hyperuricemia and the deposition of monosodium urate (MSU) crystals in the synovial fluid of articular and peri‐articular tissues, resulting in gouty arthropathy, tophi formation and organ damage (Lee, 2019). Gout is now considered a typical inflammatory disease driven by activation of the body's innate immune system, also known as an “autoinflammatory disease” (Zhao et al., 2022). In addition, immune system dysfunction plays a key role in the pathogenesis of neurodegenerative diseases (DeMaio et al., 2022). Previous studies have shown that elevated levels of uric acid linked to a reduced risk of neurodegenerative diseases such as multiple sclerosis (Rentzos et al., 2006), Parkinson's disease (Seifar et al., 2022) and AD (Du et al., 2016). Some cohort studies have suggested that hyperuricemia is associated with better cognitive function (Chen et al., 2021; Tan et al., 2023). Yet, by contrary, several epidemiological studies have shown that elevated serum uric acid was associated with cognitive impairment (Alam et al., 2020; Huang et al., 2022), especially AD (Johnson et al., 2023). Therefore, it is unclear whether gout or hyperuricemia is prone to AD or prevent against AD.

Because of the inconsistencies among studies, we conducted an up‐to‐date cohort based meta‐analysis to quantify the correlation of gout or hyperuricemia with AD.

## 2 METHODS

These systematic review and meta‐analysis were conducted in accordance with the preferred reporting items for systematic reviews and meta‐analysis (PRISMA) guidelines (Moher et al., 2009).

### 2.1 Search strategy

Two investigators (LW and Zheng Tan) independently searched the PubMed and EMBASE for papers published from their inceptions to May 20, 2023. Medical Subject Headings (MESH) terms set in the search were (“gout” OR “arthrolithiasis” OR “podagra” OR “dominus merborum” OR “hyperuricaemia”) AND (“Dementia” OR “Alzheimer's disease” OR “Mixed Dementia” OR “Cognitive decline” OR “Cognitive impairment”) NOT (“case report” OR “review” OR “meta‐analysis”).

### 2.2 Study selection

Articles were included if they fulfilled the following inclusion criteria: (1) prospective or retrospective cohort studies; (2) the exposed group consisted of gout or hyperuricemia patients, while controlled group consisted of AD patients; (3) report of risk estimates [hazard ratios (HR) or rate ratios (RR)] with 95% confidence intervals (CIs) as the outcome; (4) studies without restrictions by language of publication. Additionally, reviews, case‐control or cross‐sectional studies, and publications without adequate data were excluded.

### 2.3 Data extraction and quality assessment

The following data were extracted independently by two authors (ZT and F‐YW): first author, publication year, geographic location (country), study design, sample size, male ratio, mean or range of age among patients, diagnostic criteria of gout/ hyperuricemia and dementia, duration of follow‐up, outcome, adjustment, and study quality . The Newcastle‐Ottawa Scale (NOS) (Stang, 2010) was applied to assess the quality of the studies. Differences in the extracted data were rechecked after consultation with a third author (W‐PW) until consensus was achieved. The scale score ranged from 0 to 9 for case‐control or cohort studies. Studies with scores of <3, 4–6, and ≥7 points were considered to have a low, medium, and high quality, respectively.

### 2.4 Data synthesis and analysis

Data analysis was conducted using RevMan 5.3 and Stata 15.0 Software. We extracted the HR or RR with 95% confidence intervals (CI) from each study to evaluate the relationship between LOE and risk of dementia. Heterogeneity of HR or RR across articles was assessed using the Cochrane's *Q* and *I* ^2^ statistic; *I* ^2^ <50%, 50%∼75%, and > 75% indicated low, medium, and high homogeneity, respectively (Higgins et al., 2003). Subgroup was conducted to evaluate the possible sources of the heterogeneity. Egger's and Begg's tests were performed to assess publication bias. A two‐tailed *p* value < .05 indicated statistically significant.

## 3 RESULTS

### 3.1 Identification of studies

**FIGURE 1:**
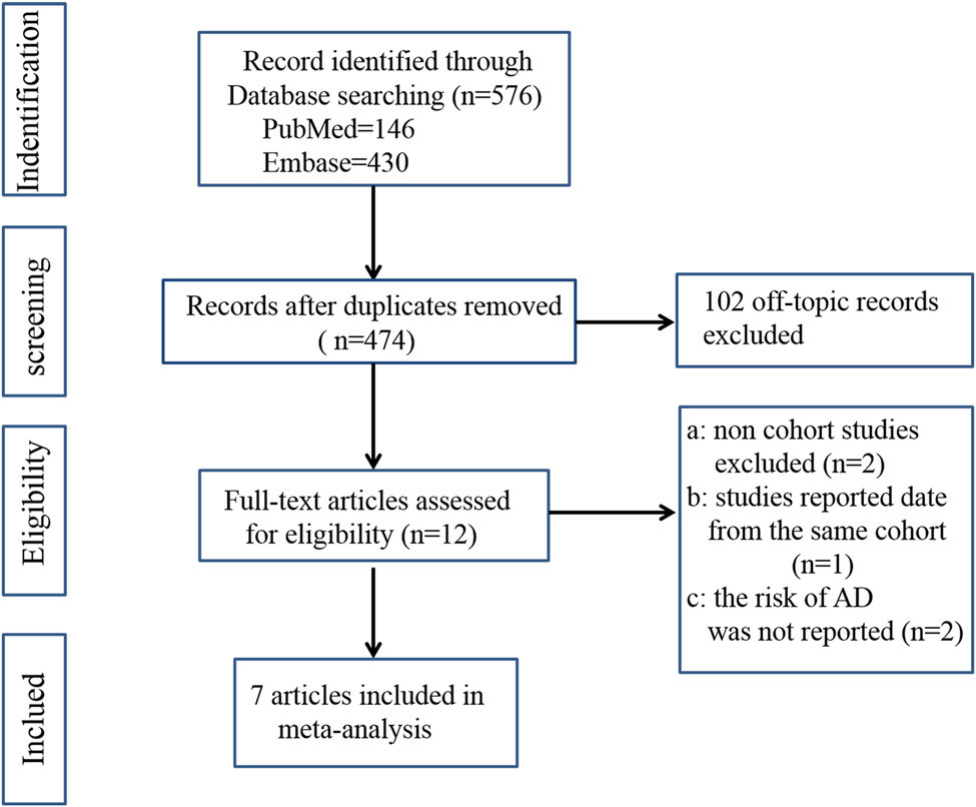
Flow diagram of the literature selection process.

As shown in Figure 1, we retrieved a total of 576 articles from our database search. Out of those, after removing duplicates, 474 articles remained and then for further filtering by their titles and abstracts. Thereafter, 12 articles were thought to be eligible and assessed for full‐text review. Seven articles were excluded, and seven research articles were included in the final meta‐analysis (Hong et al., 2015; Kim et al., 2023; Latourte et al., 2018; Lu et al., 2016; Min et al., 2021; Scheepers et al., 2019; Topiwala et al., 2023).

### 3.2 Study characteristics

The details of the included studies were shown in Table 1. Of these studies, four were conducted in Europe, while the other three in Asia. Three studies had sample sizes over 100,000, and the other four had sample size less than 50,000. Furthermore, follow‐up lasted longer than 10 years in four studies, whereas less than 10 years in other three. All studies were of high quality (NOS score ≥7).

**TABLE 1:** The characteristics of the included studies.

First author, year	Country	Study design	Sample size	Male (%)	Age (years)	Diagnosis of gout or hyperuricemia/ AD	Follow‐up (years)	Outcome RR, 95% CI	Confounders adjusted	NOS scores
Hong JY, 2015	China	Retrospective cohort	Total: 1020,755 gout: 28,779, No gout: 114,742	63	63.5 ± 9.7	ICD‐9/ICD‐9	4.3 ± 2.1	0.75 (0.61, 0.94)	Age, sex, relevant comorbidities	7
Lu N, 2016	UK	Retrospective cohort	Total: 298,029 gout: 59,224, No gout: 238,805	71	65.3 average	Read classification/ AD diagnostic codes	5 average	0.76 (0.66, 0.87)	Age, sex, entry‐time, BMI, smoking, alcohol use, physician visits, social deprivation index, comorbidities and medication use	7
Latourte A, 2018	France	Prospective cohort	Total: 1598 Hyperuricemia : 406, Non Hyperuricemia : 1192	38.3	72.9 ± 4.1	Hyperuricemia: ≥360 μmol/L for men, ≥300 μmol/L for women/ DSM‐IV NINCDS‐ADRDA	10.1 average	1.55 (0.92, 2.61);	Age, gender, tobacco and alcohol consumption, cholesterol, medical comorbidities, medication use	8
Scheepers LEJM, 2019	Sweden	Prospective cohort	Total: 1447 Hyperuricemia : 361, Non Hyperuricemia : 1086	0	47.4 ± 6.2	Hyperuricemia: ≥360 μmol/L for men, ≥300 μmol/L for women/ ICD‐8, 9, 10	44	0.77 (0.63, 0.94);	Age, sex, diabetes, hypertension, depression, head injury and CHD	8
Min KH, 2021	Korea	Retrospective cohort	Total: 135,768 gout: 22,178, No gout: 113,590	62.5	72.3 ± 6.1	ICD‐10/ICD‐10	12	0.64 (0.61, 0.68)	Age, sex, comorbidities, and average income level	7
Kim JH, 2023	Korea	Retrospective cohort	Total: 30,312 gout: 5052, No gout: 25,260	92.4	56.9 ± 13.8	ICD‐10/ICD‐10	4.8	0.73 (0.54, 0.98)	Age, sex, household income, and comorbidities	8
Topiwala A, 2023	UK	Prospective cohort	Total: 11,735	49.5	55.5 ± 8.0	Diagnostic codes/ diagnostic codes	12.4 ± 1.9	1.62 (1.30, 2.02)	Age, sex, Townsend Deprivation Index, educational qualifications, household income, historical job code, smoking, alcohol intake, waist to hip ratio, diuretic use	7

AD, Alzheimer's disease; BMI, body mass index; CAD, coronary heart disease; DSM‐IV, Diagnostic and Statistical Manual of Mental Disorders, Version IV; ICD‐9, International Classification of Diseases, Ninth Revision; ICD‐10, International Classification of Diseases, Tenth Revision; NINCDS‐ADRDA, National Institute of Neurological and Communicative Disorders and Stroke‐Alzheimer's Disease and Related Disorders Association.

### 3.3 The effect of gout or hyperuricemia on AD

**FIGURE 2:**
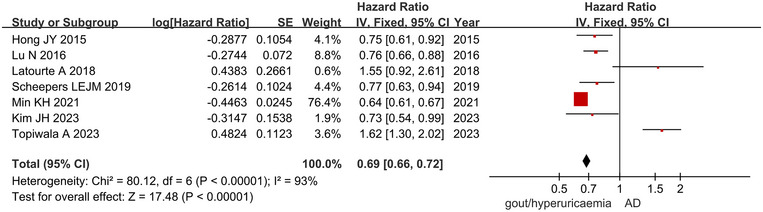
Forest plot presenting effect estimates for the risk of AD in patients with gout or hyperuricemia.

Pooled results showed that individuals presenting with gout or hyperuricemia had a high probability of reducing AD (RR = 0.69, 95%CI: 0.66∼0.72; Figure 2). There was a moderate risk of heterogeneity among the studies (*I* ^2^ = 93%, *p* < .001).

### 3.4 Subgroup analysis

In the subgroup analysis for the RR of AD with gout or hyperuricemia patients, including study design, sample size, location, and adjustment for confounding factors indicated consistent results (*p* for subgroup differences all > .05). Neither subgroup analysis by study design nor sample size could explain the origin of the higher heterogeneity. However, when using regional distribution as subgroup, the Asia studies showed lower heterogeneity (*I* ^2^ = 28%, Table 2). This indicated that regional distribution was the source of heterogeneity.

**TABLE 2:** Subgroup analysis for the risk ratio of AD in patients with gout/hyperuricemia.

			Heterogeneity (*I* ^2^, Tau^2^ and *p* value)		
Subgroups	Included studies	RR(95% CI)	*I* ^2^ (%)	Tau^2^	*p* Value
Study design					
Prospective cohort	3	1.11 (0.96∼1.28)	92.0	25.67	.16
Retrospective cohort	4	0.66 (0.63∼0.69)	59.0	7.29	.06
Sample size					
≥50,000	3	0.66 (0.63∼0.69)	71.0	6.81	.03
<50,000	4	1.03 (0.90∼1.17)	91.0	31.70	.68
Regional distribution					
Europe	4	0.92 (0.83∼1.01)	92.0	39.28	.09
Asia	3	0.65 (0.62∼0.68)	28.0	2.78	.25

### 3.5 Publication bias

**FIGURE 3:**
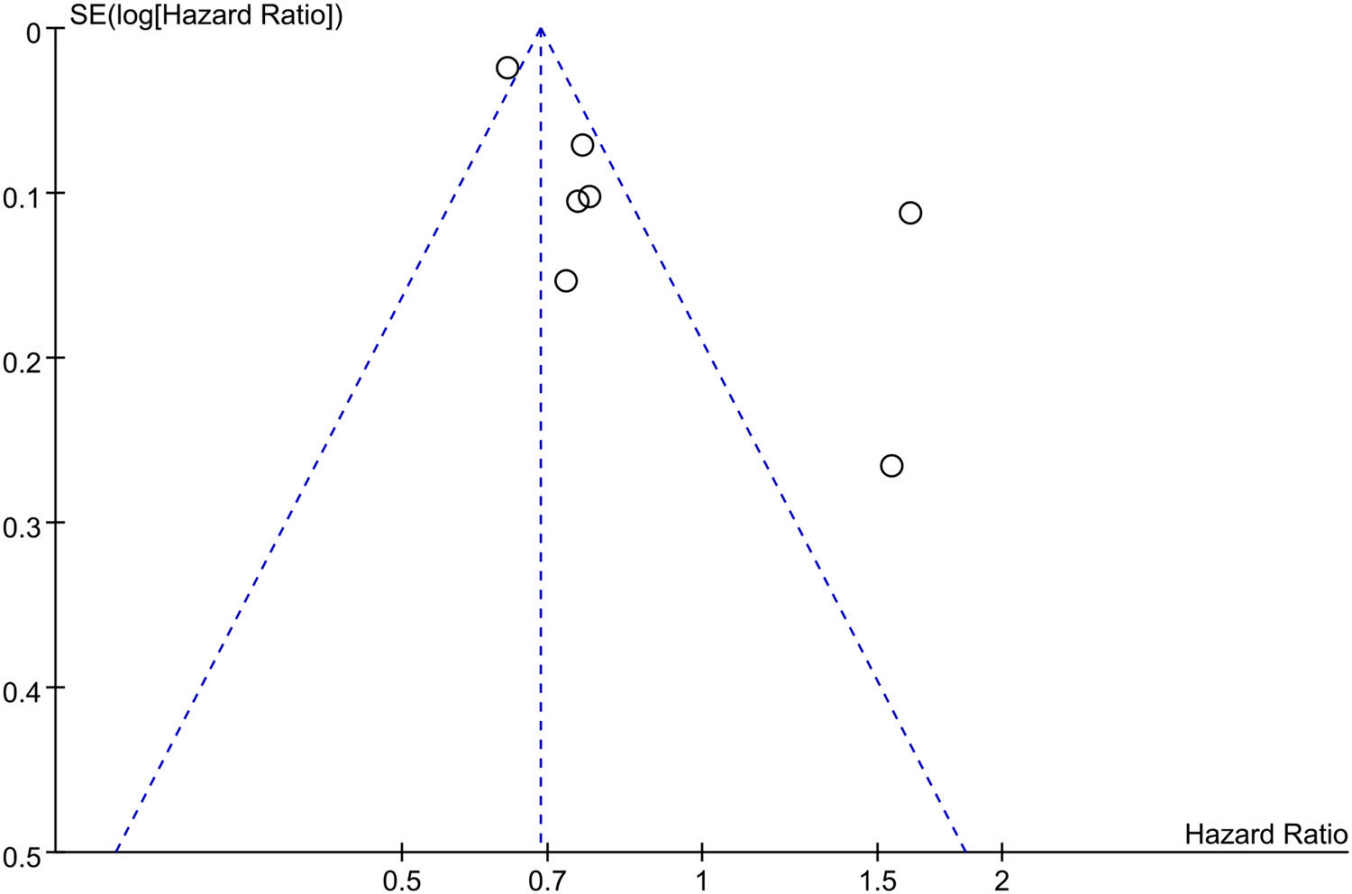
Funnel plot for AD in patients with gout or hyperuricemia.

On visual inspection, the funnel plot seemed to be asymmetrical (Figure 3). However, both the Begg's test (*t* = −1.50, *p* = .133) and Egger's test (*t* = −0.51, *p* = .633) did not reveal potential publication bias.

## 4 DISCUSSION

In the current meta‐analysis, we comprehensively evaluated the association between gout/hyperuricemia and AD based on evidence from high‐quality cohort studies. Interestingly, the results showed a 31% AD risk reduction for gout or hyperuricemia. This indicated that gout or hyperuricemia might be a protective factor approach to prevent AD formation. Additionally, these relationships remained consistent across subgroup and sensitivity analyses.

Although a previous meta‐analysis investigated the association between gout/hyperuricemia and dementia, the results suggested no statistically significant risk of all‐cause dementia in patients with gout or hyperuricemia (Pan et al., 2021). In other words, it did not imply that gout or hyperuricemia was free of risk for all types of dementia. The recognized evidence is that gout or hyperuricemia is a risk factor for stroke and closely related to the occurrence of vascular dementia (Cipolletta et al., 2022; Serdarevic et al., 2020). The lack of subgroup analysis due to the limited inclusion of studies may be a reasonable explanation for the negative conclusion in that meta‐analysis. In addition, a recent meta‐analysis by Li et al. (2023) included four primary studies, exploring the relationship between gout and the risk of AD, but omitted three other important studies that may have reduced the statistical power. By contrast, in the current analysis, we included more recent studies and analyzed data on the basis of subgroups, thus providing potential evidence for the association between gout/hyperuricemia and AD.

The role of gout or hyperuricemia in the pathogenesis of AD has often been described as controversial (Singh, 2018). As mentioned above, our study showed a negative correlation between gout/hyperuricemia and AD. However, there were some previous studies with totally different conclusion: reporting that gout or hyperuricemia had no correlation with AD or even a positive association (describing gout or hyperuricemia as a risk factor) (Lai et al., 2013; Lee, 2019; Singh & Cleveland, 2018). Evidence that links chronic inflammation to neurodegenerative processes in AD is accumulating. Persistent systemic inflammation in patients with hyperuricemia or gout can also affect the inflammatory response of brain immune cells (Wang et al., 2021; Zhao et al., 2022). On the one hand, activated astrocytes play a vital neuroprotective role by inhibiting senile plaques (SPs) aggregation and Aβ‐amyloid deposition (Singh, 2022); on the other hand, they also promote the release of reactive oxygen species (ROS) and inflammatory cytokines in turn (Bai et al., 2022). In addition, superoxide enzymes produced in uric acid formation also increase the oxidative stress response (Liu et al., 2021). Thus, oxidative stress may act as a bridge connecting the pathogenesis of hyperuricemia and AD, at least partially explaining the increased risk of AD in elderly gout or hyperuricemia patients.

However, if we accept that gout or hyperuricemia leads to risk reduction, what is the reason or mechanism behind this reverse association? A vital mechanism may be the dual effects of uric acid on prooxidation and antioxidation (Kang & Ha, 2014; Ndrepepa, 2018), but there are few studies comparing the strength of their interactions for circulating uric acid metabolism. As antioxidants and free radical scavenger, uric acid also plays a neuroprotective role by alleviating oxidative stress damage induced by aging or inflammation (Tana et al., 2018). In addition, mitochondrial dysfunction has been confirmed as one of the pathogenesis of AD (Perez Ortiz & Swerdlow, 2019), while uric acid preserves mitochondrial function by inhibiting the cytotoxic effect of lactoperoxidase and repairing DNA damaged by oxygen radical (Johnson et al., 2023; Yu et al., 1998).

Recent clinical studies have found that younger older adults (65–79 years) may be more helpful in exposing the cognitive effects of high uric acid (Euser et al., 2009). This is consistent with our meta‐analysis results, as the initial average age at which gout or hyperuricemia was diagnosed in the most of included studies ranged from 60 to 80 years old. It has been speculated that elevated uric acid in midlife may promote cognitive reserve but subsequent long‐term chronic elevation could induce its prooxidant property (Alam et al., 2020).

For the subgroup analysis, the source of heterogeneity may be related to regional distribution. Therefore, more studies from different regions are needed to clarify the connection of gout or hyperuricemia with risk of AD.

Some limitations should be addressed. First, as mentioned above, there are the limited studies that examine the effect of gout or hyperuricemia on AD, thereby reducing the accuracy of the pooled estimates. Additionally, it is not possible to distinguish the different effects of gout on various types of dementia. Second, inconsistencies in the clinical diagnostic criteria for gout, hyperuricemia and AD across studies may affect the stability of outcomes. Third, previous studies have shown that the use of antigout preparations, with particular reference to benzbromarone, was negatively correlated with dementia (Taipale et al., 2018). Since statistical adjustments in numerous studies did not include drugs use, it is possible that the risk of dementia in gout or hyperuricemia patients was underestimated. Finally, although most of included studies employed maximum adjusted risk estimates for potential factors, residual confounding of unmeasured factors would persist, which is an inherent limitation of clinical research.

In summary, our meta‐analysis suggested negative relationship of gout or hyperuricemia with risk of AD. This finding supports the protective effect of gout or hyperuricemia on cognitive function. More basic and clinical evidence from future studies needs to be clarified the cause‐and‐effect association between gout and AD.

## AUTHOR CONTRIBUTIONS

J‐CW were responsible for study design, overall planning, and project administration. LW and ZT contributed to the data curation. ZT, F‐YW, and W‐PW performed investigation, methodology, resources, and validation. LW and ZT drafted the original manuscript. ZT contributed to statistical analysis and interpreted the results. All authors have read and approved the submission.

## CONFLICT OF INTEREST STATEMENT

The authors declare no conflict of interest in this paper.

### PEER REVIEW

The peer review history for this article is available at https://publons.com/publon/10.1002/brb3.3207.

## Data Availability

The data that support the findings of this study are available from the corresponding authors upon reasonable request.
